# *Bacillus cereus*-induced food-borne outbreaks in France, 2007 to 2014: epidemiology and genetic characterisation

**DOI:** 10.2807/1560-7917.ES.2016.21.48.30413

**Published:** 2016-12-01

**Authors:** Benjamin Glasset, Sabine Herbin, Laurent Guillier, Sabrina Cadel-Six, Marie-Léone Vignaud, Joel Grout, Sylvie Pairaud, Valérie Michel, Jacques-Antoine Hennekinne, Nalini Ramarao, Anne Brisabois

**Affiliations:** 1 Université Paris-Est, ANSES, Laboratory for Food Safety, Maisons-Alfort Cedex, France; 2 Micalis Institute, INRA, AgroParisTech, Université Paris-Saclay, Jouy-en-Josas, France; 3 ACTALIA, La Roche sur Foron, France; 4 These authors contributed equally to this work

**Keywords:** Bacillus cereus, epidemiology, food-borne infections, outbreak, virulence factors, genotyping

## Abstract

The aim of this study was to identify and characterise *Bacillus cereus* from a unique national collection of 564 strains associated with 140 strong-evidence food-borne outbreaks (FBOs) occurring in France during 2007 to 2014. Starchy food and vegetables were the most frequent food vehicles identified; 747 of 911 human cases occurred in institutional catering contexts. Incubation period was significantly shorter for emetic strains compared with diarrhoeal strains A sub-panel of 149 strains strictly associated to 74 FBOs and selected on Coliphage M13-PCR pattern, was studied for detection of the genes encoding cereulide, diarrhoeic toxins (Nhe, Hbl, CytK1 and CytK2) and haemolysin (HlyII), as well as *panC* phylogenetic classification. This clustered the strains into 12 genetic signatures (GSs) highlighting the virulence potential of each strain. GS1 (*nhe* genes only) and GS2 (*nhe*, *hbl* and *cytK2*), were the most prevalent GS and may have a large impact on human health as they were present in 28% and 31% of FBOs, respectively. Our study provides a convenient molecular scheme for characterisation of *B. cereus* strains responsible for FBOs in order to improve the monitoring and investigation of *B. cereus*-induced FBOs, assess emerging clusters and diversity of strains.

## Introduction

The *Bacillus cereus* sensu lato group includes the following closely related spore-forming species: *B. cereus* sensu stricto*, B. thuringiensis, B. cytotoxicus, B. weihenstephanensis, B. mycoides, B. pseudomycoides* and *B. anthracis* [1]. The first four species are known to be involved in food poisoning [1]. *B. thuringiensis* is also mainly known as a biopesticide due to production of insecticidal toxins [2]. *B. anthracis* is highly virulent in mammals and is the causative agent of anthrax [3]. *B. cytotoxicus* is a newly identified group of strains that induce severe food poisoning. They are characterised by the production of cytotoxin K-1 (CytK-1) and a relatively high genomic diversity compared with other *B. cereus* strains [1].

*B. cereus* is currently the second most frequently found causative agent of confirmed and suspected food-borne outbreaks (FBOs) in France after *Staphylococcus aureus* [4]. Depending on the evidence implicating a food vehicle source during epidemiological and microbiological FBO investigations, the outbreaks are referred as a strong-evidence or weak-evidence FBO. Briefly, an FBO is defined as ‘strong-evidence’ when the following information is known and reported: food vehicle, food source, the link between outbreak cases and the food vehicle, place of exposure, and contributory factors. When several parts of the information are missing, the FBO is considered as ‘weak-evidence’ FBO [5].

Between 2006 and 2014 in France, *B. cereus* was recorded as the second or third major cause in weak-evidence FBOs. In 2014, *B. cereus* represented the second cause in weak-evidence FBOs, with 1,902 human cases for 224 FBOs, and the second cause of strong-evidence FBOs, with 23 FBOs accounting for 447 human cases and 18 hospitalisations [4]. The increase in *B. cereus*-induced FBOs is partly due to the input of national health and food safety authorities in the epidemiological and microbiological investigations of suspected FBOs. Indeed, *B. cereus* strains isolated from foodstuff suspected of being involved in an FBO are now usually collected by the laboratory for food safety in ANSES. To illustrate this, during 1996 to 2005, only 94 strong-evidence and 196 weak-evidence FBOs were reported, whereas for 2014 alone, 23 and 241 strong- and weak-evidence FBOs were notified, respectively showing the high input of the authorities. Nevertheless, the number of total human *B. cereus* cases is likely to be underestimated because individuals with gastrointestinal infections rarely seek medical advice and if they do, stools sample are not always asked for by physicians.

*B. cereus* can induce two types of gastrointestinal disease, leading to emetic or diarrhoeal syndromes. The symptoms associated with *B. cereus* infection are generally mild and self-limiting, but more serious and even fatal cases have been described in France and around the world [6]. The emetic syndrome is characterised by vomiting and nausea, usually 30 minutes to 6 hours after ingestion, and can be confused with FBOs caused by *Staphylococcus aureus*. This syndrome is due to the ingestion of a thermostable toxin known as cereulide, pre-formed in food before ingestion of contaminated foods. The emetic *B. cereus* strains represent a cluster of strains characterised by the presence of the plasmid-located *ces* gene encoding an enzyme involved in cereulide synthesis [7].

Diarrhoeic symptoms are characterised by abdominal cramps and watery diarrhoea within 8 to 16 hours after ingestion of contaminated foods. These diarrhoeal symptoms and incubation periods can be easily confused with those caused by *Clostridium perfringens* food poisoning. More precise information about diarrhoeic strains is thus necessary to discriminate between possible causative agents and allow better diagnosis during FBOs. The diarrhoeal syndrome occurs after ingestion of vegetative cells or spores of diarrhoeic strains. This syndrome is generally attributed to at least three enterotoxins: haemolysin BL (Hbl), which has three components B, L_1_ and L_2_; non-haemolytic enterotoxin (Nhe) with its three components Nhe-A, Nhe-B and Nhe-C, and cytotoxin K (CytK). Two forms of cytotoxin K have been described, CytK-1 and CytK-2, the former being more cytotoxic than the latter [8]. In addition, *B. cereus* produces other toxins such as haemolysin II (HlyII), metalloproteases such as InhA1 and InhA2, and the cell wall peptidase FM (CwpFM), which may also be involved in pathogenicity [9-11]. The pathogenic spectrum of *B. cereus* ranges from strains used as probiotics to strains that are lethal to humans and it remains difficult to predict the pathogenic potential of a strain. Apart from strains encoding *ces* or *cytK-1* genes, which are virulent and well described in the literature [8,12], the pathogenicity of *B.cereus* diarrhoeal strains is not fully understood and there are currently no specific markers to unambiguously differentiate between pathogenic and harmless strains. Indeed, the genetic studies carried out to date have been inconclusive and, regardless of the diseases they cause, all strains seem to carry genes encoding at least one of the known diarrhoeal toxins [13]. However, highly toxic strains do not necessarily overproduce these toxins [14]. The aim of this study was therefore to identify and characterise *B. cereus* strains from a unique national collection of 564 strains strongly related to 140 FBOs that occurred in France during 2007 to 2014 in order to improve the monitoring and investigation of *B. cereus*-induced FBOs, assess the risk of emerging clusters of strains and identify strain variability.

## Methods

### Epidemiological data

The epidemiological data related to each FBO were mainly collected through interviews or questionnaires by local health authorities. The suspected food in each FBO was traced by the local services of the French Ministry of Agriculture and Food (DDPP, Department for protection of populations). Collected data included a record of the type of suspected food, preparation location and date, type of packaging, number of human cases, symptoms and incubation periods. Then, a database of ANSES (French Agency for Food, Environmental and Occupational Health and Safety) was built, gathering epidemiological data as well as analytical results of *B. cereus* enumeration in food, strain characterisation and toxin production.

### Strain collection

For each FBO, all bacterial strains from suspected food were isolated by plating leftovers on selective media plates allowing the discrimination of *B. cereus* from other bacterial pathogens (*S. aureus, C. perfringens*, etc). Identification and numeration of one to five *B. cereus* strains per FBO were conducted by plating the strains on selective *B. cereus* agar media (MYP agar media: mannitol-phenol red-egg yolk medium (Biokar) according to the International Organization for Standardization (ISO) 7932 standard method or BACARA (BioMérieux), previously certified commercial alternative method (AES 10/10–07/10). All isolates were tested for haemolytic activity on sheep blood agar [15], lecithinase production on MYP agar media and starch hydrolysis on plate count agar (BioMérieux).

### DNA extraction

DNA was extracted after overnight incubation of the strains at 30 °C on trypticase soy agar with 0.6% yeast extract (Sigma-Aldrich) using the DNeasy Blood and Tissue Kit (Qiagen). DNA was quantified by absorbance at 260 nm on a Nanodrop1000 spectrophotometer (Thermo scientific).

### Coliphage M13 sequence-based PCR typing

To study strain diversity and discriminate between strains isolated in samples within the same FBO, *B. cereus* strains were typed using coliphage M13 sequence-based PCR (M13-PCR) derived from an RAPD technique and adapted from [16]. The PCR mix contained 40 ng of DNA template, 0.9 mM dNTP mix (Roche Diagnostics), 4 mM MgCl_2_, 2 µM primer (GAGGGTGGCGGCTCT), 2.5 U Goldstar DNA polymerase, and Goldstar buffer (Eurogentec). Thermal cycling using the Veriti Thermal Cycler (Applied Biosystems) included a denaturation step at 94 °C for 3 min, followed by 35 cycles of 1 min at 94 °C, 1 min at 40 °C, 8 min at 68 °C and an elongation step at 68 °C for 8 min. The amplified DNA was analysed by SDS-PAGE electrophoresis. The M13-PCR patterns were visualised using ChemiDoc XRS imaging system. Then, DNA profiles were analysed with BioNumerics 7.1 software (Applied Maths).

### *panC* gene sequencing

*B. cereus* strains were assigned to the seven known phylogenetic groups according to partial sequencing of the *panC* gene [17]. The sequencing was carried out by a commercial facility (Eurofins MWG Operon). The classification into the phylogenetic groups was performed using the algorithm described in [17]. The two typing methods *panC* gene sequencing and M13-PCR typing were used for separate objectives. This study did not explore the correlation between the two methods.

### Virulence gene detection

The presence of potential virulence genes *cytK-1, cytK-2, hblA, hblC, hblD, nheA, nheB, nheC, hlyII* and *ces* [10,13] was evaluated by PCR. As the genetic diversity of *B. cytotoxicus* strains possessing *cytK-1* is substantial, the primers used to detect the other virulence genes were not suitable for those particular strains. The PCR was performed with the Veriti Thermal Cycler. The final reaction mixture (25 µL) contained 200 µM dNTPs, 1X PCR buffer, 1 U FastStart Taq DNA Polymerase (Roche), 200–1,000 nM primers, and 2 µL (ca 10 ng) template DNA. The amplification protocol comprised initial denaturation at 94 °C for 5 min followed by 30 cycles of 94 °C for 30 s, 58 °C for 60 s, and 72 °C for 90 s and final extension at 72 °C for 7 min. PCR products were analysed by SDS-PAGE electrophoresis.

### Enterotoxin quantification

The production of the enterotoxins Nhe and Hbl was tested using two immunological tests, the BCET-RPLA Toxin detection kit (Oxoïd) and Tecra kit (BDE VIA, 3M-Tecra), respectively, after culture in brain heart infusion broth (Biomérieux) for 6 hours at 30 °C with stirring [18].

### Database and statistical analysis

Strain characterisation results and epidemiological data were entered into a central database using BioNumerics software. The distribution of mean incubation periods, i.e. the time between ingesting contaminated food and symptom onset, was characterised using R 3.1 software and the ‘fitdistrplus’ package [19]. The log-normal was fitted to data according to maximum-likelihood estimation. To study seasonal variation in the occurrence of FBOs, the distribution of FBO dates was analysed throughout the year according to a previously described method [20].

## Results

### Epidemiological and clinical data

We studied a collection of 564 *B. cereus* strains associated with 140 FBO that occurred in France during 2007 to 2014. In 66 of the FBOs, *B. cereus* was isolated concomitantly with other bacterial species (including *S. aureus* and *C. perfringens*) during microbiological investigations, making it impossible to affirm that *B. cereus* was the cause of these FBOs. Our study therefore focused on 339 *B. cereus* strains isolated from food samples analysed during 74 FBOs where no other pathogenic bacteria were detected in the food during microbiological investigations (Table 1). These 74 FBOs resulted in 911 human cases. Data on sex and age of the cases were not always available and could therefore not be included in the study.

**Table 1:** Epidemiological and microbiological data of food-borne outbreaks associated solely with *Bacillus cereus*, France, 2007–2014 (74 outbreaks, 339 strains)

FBO	Year	Incriminated food	Human cases n	Incubation period in hours	Symptoms	Strain patterns identified n	Outbreak setting^a^	CFU/g	Genetic signature
1	2007	Semolina	5	0–3	Vomiting	1	Commercial catering	1.20E + 07	GS3
2	2007	Shrimp	12	21–24	Vomiting, diarrhoea	1	Commercial catering	6.80E + 04	GS1
3	2007	Tomatoes	4	0–3	Vomiting, diarrhoea	1	Commercial catering	7.00E + 02	GS4
4	2008	Semolina	40	12–15	Diarrhoea	1	Staff canteen	1.20E + 03	GS1
5	2008	Tabbouleh and minced beef	NK	NK	NK	1	Commercial catering	5.00E + 03	GS2
6	2008	Mixed salad, goulash mixed beef and mashed potatoes	19	NK	Vomiting, diarrhoea	4	Medico-social institute	6.00E + 02	GS1; GS2; GS7; GS12
7	2008	Mashed potatoes and boiled potatoes	28	NK	Vomiting, diarrhoea	2	Medico-social institute	9.20E + 05	GS7; GS8
8	2008	Mixed salad (rice and corn)	2	NK	Abdominal pains, vomiting	1	Staff canteen	1.90E + 03	GS2
9	2008	Rice salad	13	12–15	Abdominal pains, vomiting, other	1	Medico-social institute	2.00E + 03	GS2
10	2008	Semolina	61	3–6	Abdominal pains, vomiting	1	School canteen	1.00E + 04	GS7
11	2008	Semolina and lamb	4	0–3	Vomiting	1	Commercial catering	5.50E + 04	GS3
12	2008	Mashed potatoes, mashed celery, roast pork, sauce and pasta	5	6–9	Diarrhoea	2	Medico-social institute	1.50E + 05	GS4; GS7
13	2008	Cream caramel and smoked salmon	11	9–12	Diarrhoea, other	3	Commercial catering	3.00E + 03	GS2; GS8
14	2008	Fruit salad	70	NK	NK	1	Staff canteen	6.30E + 03	GS3
15	2008	Tandoori chicken	10	6–9	Vomiting, diarrhoea	2	Commercial catering	4.60E + 03	GS6
16	2008	Wheat	3	9–12	Diarrhoea	3	Commercial catering	1.60E + 06	GS1; GS4
17	2009	Tiramisu	15	0–3	Vomiting, diarrhoea	1	Company canteen	8.00E + 02	GS9
18	2009	Fish in coconut milk	2	0–3	Nausea, other	1	Commercial catering	1.10E + 04	GS1
19	2009	Mashed potatoes	24	NK	Vomiting, diarrhoea	1	School canteen	4.00E + 02	GS7
20	2009	Cantonese rice	2	0–3	Vomiting, other	1	Family	1.60E + 05	GS3
21	2009	Mashed potatoes, roast beef and French beans	7	6–9	Vomiting, diarrhoea	3	Medico-social institute	1.90E + 03	GS3; GS5
22	2009	Quenelle of pike	15	0–3	Vomiting, diarrhoea, other	1	Staff canteen	1.20E + 03	GS6
23	2009	Sandwich (tomato, carrots, chicken)	7	0–3	Abdominal pains, nausea	4	Commercial catering	5.00E + 03	GS1; GS2; GS6; GS10
24	2009	Chicken sauce	15	NK	Vomiting,- diarrhoea	1	Commercial catering	5.00E + 02	GS3
25	2009	Squid sauce	3	9–12	Diarrhoea	1	Staff canteen	2.10E + 05	GS12
26	2009	Sauteed shrimp	4	0–3	Vomiting, diarrhoea	7	Commercial catering	1.90E + 04	GS1; GS4; GS6
27	2009	Semolina and peas	7	3–6	Nausea, diarrhoea, other	5	Staff canteen	2.00E + 07	GS2; GS5
28	2010	Salad	44	NK	Vomiting, diarrhoea, other	3	School canteen	1.00E + 03	GS2
29	2010	Pasta gratin	2	0–3	vomiting - diarrhoea	1	Family	1.50E + 07	GS3
30	2010	Sausage and rice salad	8	0–3	Vomiting, diarrhoea	1	Family	3.00E + 03	GS3
31	2010	Paella	27	6–9	Diarrhoea	1	Medico-social institute	2.80E + 04	GS2
32	2010	Samosa and marinated shrimp tail	3	0–3	Diarrhoea	13	Commercial catering	2.90E + 05	GS1; GS2; GS4; GS5; GS6; GS10
33	2010	Chicken	8	3–6	Vomiting, diarrhoea	1	Family	6,50E + 04	GS3
34	2010	Tabbouleh	11	NK	Abdominal pains, other	1	Medico-social institute	NK	GS2
35	2010	Mashed potatoes and mashed vegetables	19	NK	Vomiting, diarrhoea, other	1	Medico-social institute	1.20E + 04	GS1
36	2010	Pasta salad and rice salad	20	0–3	Vomiting, diarrhoea	7	Family	9.60E + 07	GS1; GS3; GS4; GS5; GS6
37	2011	Mixed dish, soup, mixed ham, mixed apple and lasagne bolognese	19	6–9	Vomiting, diarrhoea	2	Medico-social institute	3.10E + 03	GS3
38	2011	Shrimp	3	0–3	Abdominal pains, vomiting, other	2	Commercial catering	1.90E + 03	GS1
39	2011	Moussaka	1	3–6	Abdominal pains	3	Commercial catering	8.20E + 04	GS1; GS4; GS5
40	2011	Spaghetti	18	12–15	Vomiting, diarrhoea	2	School canteen	1.00E + 03	GS8
41	2011	Couscous, semolina, lamb, vegetable dish	19	9–12	Nausea, diarrhoea	2	Medico-social institute	2.30E + 03	GS4; GS11
42	2011	Carrots	3	3–6	Vomiting, diarrhoea, other	1	Commercial catering	5.80E + 03	GS2
43	2011	Mashed potatoes	10	NK	Vomiting, diarrhoea	1	School canteen	7.80E + 04	GS4
44	2011	Mashed celery	15	12–15	Vomiting, diarrhoea	1	Staff canteen	1.00E + 05	GS7
45	2011	Tomatoes and fish	3	12–15	Vomiting, diarrhoea	1	Medico-social institute	5.50E + 03	GS2
46	2011	Miso soup	1	NK	NK	1	Family	1.50E + 03	GS9
47	2011	Mixed salad	3	0–3	Vomiting, diarrhoea	1	Medico-social institute	2.00E + 03	GS2
48	2011	Tomato, corn, courgette dish	9	6–9	Abdominal pains, vomiting	1	School canteen	4.00E + 03	GS2
49	2011	Samosa	9	0–3	Nausea, other	1	Commercial catering	1.,00E + 09	GS6
50	2011	Rice and shellfish dish and fish	6	3–6	Abdominal pains, nausea	2	Staff canteen	2.70E + 03	GS5; GS6
51	2012	Apricot compote, mashed carrots and mashed broccoli	8	9–12	Vomiting	1	School canteen	7.00E + 02	GS1
52	2012	Paella	2	0–3	Vomiting, diarrhoea, other	3	Commercial catering	2.10E + 04	GS1; GS3; GS10
53	2012	Pasta	60	0–3	Vomiting, diarrhoea	3	School canteen	5.80E + 04	GS5
54	2012	Mixed salad	8	18–21	Abdominal pains, vomiting, other	1	Family	4.00E + 02	GS2
55	2012	Chicken	NK	NK	Other	3	Commercial catering	4.00E + 03	GS2; GS5
56	2012	Lamb meat	5	6–9	Vomiting, diarrhoea	1	Staff canteen	2.30E + 03	GS2
57	2012	Mashed fish	18	9–12	Vomiting, diarrhoea	1	Medico-social institute	4.00E + 02	GS7
58	2012	Diced mixed vegetables	14	9–12	Vomiting, diarrhoea	1	Medico-social institute	4.00E + 02	GS2
59	2012	Millefeuille pastry	2	3–6	Nausea	1	Commercial catering	2.00E + 03	GS2
60	2012	Onion soup	5	9–12	Vomiting	1	School canteen	4.00E + 02	GS2
61	2013	Semolina	3	3–6	Vomiting, diarrhoea	2	Family	1.00E + 04	GS5; GS10
62	2013	Grilled pork	2	6–9	Vomiting, diarrhoea	2	Family	1.80E + 04	GS1; GS9
63	2013	Cheese-topped dish of seafood, pasta	15	6–9	Diarrhoea, other	4	Staff canteen	6.50E + 03	GS1; GS3; GS4
64	2013	Mashed potatoes	12	3–6	Vomiting, diarrhoea, other	2	Medico-social institute	2.90E + 03	GS1; GS3
65	2013	Pineapple	5	NK	Other	2	School canteen	4.50E + 02	GS1; GS9
66	2013	Mashed spinach	13	6–9	Vomiting, diarrhoea	3	Medico-social institute	1.00E + 04	GS1; GS4
67	2013	Vegetable soup	10	15–18	Vomiting, diarrhoea	1	Medico-social institute	9,10E + 02	GS2
68	2013	Mixed salad	NK	6–9	Abdominal pains	1	School canteen	5.50E + 02	GS2
69	2013	Spinach	8	0–3	Vomiting, diarrhoea, other	2	Staff canteen	3.60E + 02	GS5; GS10
70	2013	Mixed pie	19	12–15	Vomiting, diarrhoea	1	Medico-social institute	4.00E + 02	GS1
71	2014	Mashed parsnips	11	0–3	Vomiting	2	School canteen	4,00E + 02	GS3
72	2014	Shrimp	6	0–3	Abdominal pains, vomiting	2	School canteen	7.70E + 03	GS1
73	2014	Polenta	25	18–21	Abdominal pains, diarrhoea	1	Medico-social institute	9.00E + 03	GS5
74	2014	Semolina and ginger (spice)	11	0–3	Vomiting, diarrhoea	2	Family	1.50E + 06	GS3; GS6

FBO: food-borne outbreak; NK: not known.

^a^ Medico-social institutes included centres for disabled people, leisure centres, retirement homes and other community facilities.

**Figure 1:**
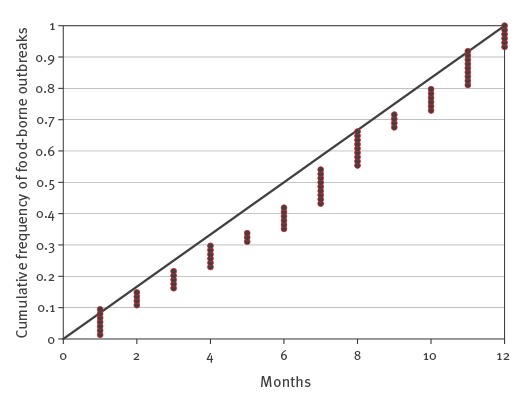
Distribution of food-borne outbreaks associated to *Bacillus cereus* by month of outbreak compared to a theoretical uniform distribution, France, 2007–2014

Over the eight years of the survey, the occurrence of FBOs was not subject to any seasonal effect (Figure 1). Emetic and diarrhoeal symptoms of human cases were often present at the same time and were reported for 57% of FBOs (42/74), whereas abdominal pains, diarrhoeic or emetic syndromes alone occurred in 4% (36/911), 12% (109/911) and 13% (118/911) total human cases, respectively.

For the theoretical uniform distribution, each dot represents a food-borne outbreak.

**Figure 2:**
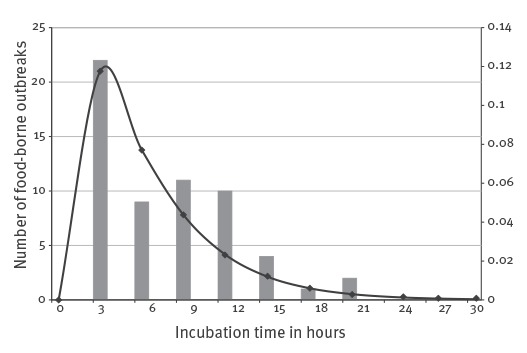
Distribution of food-borne outbreaks by incubation periods for the entire *Bacillus cereus* collection, France, 2007–2014

Between 400 and 10^8^*B. cereus* CFU/g were found in the incriminated foods. Levels lower than 10^5^ CFU/g were observed in 48/57 FBOs due to diarrhoeal strains and in 11/17 FBOs due to emetic strains (Table 1). The incubation period (time between ingestion of contaminated food and symptom onset) varied from less than 3 hours to 21 hours (Figure 2). The mean incubation period was 5.7 hours (standard deviation (SD) 1.3) and could vary within the same FBO (Table 1). However, the incubation period was significantly shorter for emetic strains (carrying the *ces* gene) – mean: 2.6 hours (SD: 2.1) – compared with diarrhoeal strains (mean: 6.6 hours (SD: 1.4).

Bars represent the number of food-borne outbreaks for each incubation time.

A single food source was incriminated for 57% of FBOs (42/74), of which 14/42 were associated with starchy food, 8/42 and 7/42 FBOs with vegetables and with mixed dishes composed of starchy food or vegetables, respectively (Table 1). Only 14% (10/74) of FBOs were associated with foodstuffs of animal origin.

Furthermore, 60% of FBOs (44/74) occurred in institutional catering, involving 82% (747/911) of the human cases. FBOs were poorly reported in a family context, which represented 13% of the FBOs (10/74) and 7% (64/911) of the human cases (Table 1). The remaining 27% (20/74) of FBOs occurred in a commercial catering context, involving 11% (100/911) of cases.

### Strain characterisation

Phenotypic analysis of the strains showed that 92% (312/339) of the strains produced lecithinase. Haemolytic activity on sheep blood agar was detected for 87% (295/339). Some 48% (163/339) of strains were able to hydrolyse starch (data not shown). The *panC* gene sequences were used to assign *B. cereus* strains to one of the seven previously described phylogenetic groups I to VII (Table 2). Group I was not represented in the strains analysed. Group III was the most represented (46%; 156/339). Groups IV and II represented 24% (81/339) and 19% (64/339), respectively. The distribution of strains in groups VII, VI and V were 5% (17/339), 4% (14/339) and 2% (7/339), respectively.

**Table 2:** Genetic signatures of *Bacillus cereus* strains according to gene detection and panC phylogenetic groups, France, 2007–2014 (n = 159)

Genetic signature	Number of strains	Genes detected						*panC* phylogenetic groups
		*cytk-1*	*cytk-2*	*ces*	*hlyII*	*nheABC*	*hblCDA*	
GS1	34	Neg	Neg	Neg	Neg	Pos	Neg	II -III - IV
GS2	28	Neg	Pos	Neg	Neg	Pos	Pos	IV
GS3	25	Neg	Neg	Pos	Neg	Pos	Neg	III
GS4	18	Neg	Pos	Neg	Neg	Pos	Neg	II - III
GS5	18	Neg	Neg	Neg	Pos	Pos	Pos	II - III
GS6	10	Neg	Pos	Neg	Pos	Pos	Pos	II - IV
GS7	8	Pos	ND	ND	ND	ND	ND	VII
GS8	6	Neg	Neg	Neg	Neg	BC	AD	VI
GS9	4	Neg	Pos	Neg	Pos	Pos	Neg	II - III
GS10	5	Neg	Neg	Neg	Neg	Pos	Pos	IV - V
GS11	1	Neg	Pos	Pos	Neg	Pos	Neg	III
GS12	2	Neg	Neg	Neg	Pos	Pos	Neg	II

AD: only *hblA* and *hblD* detected; BC: only *nheB* and *nheC* detected; ND: primers used are unable to detect these genes in GS7 group strains; Neg: negative; Pos: positive.

**Figure 3:**
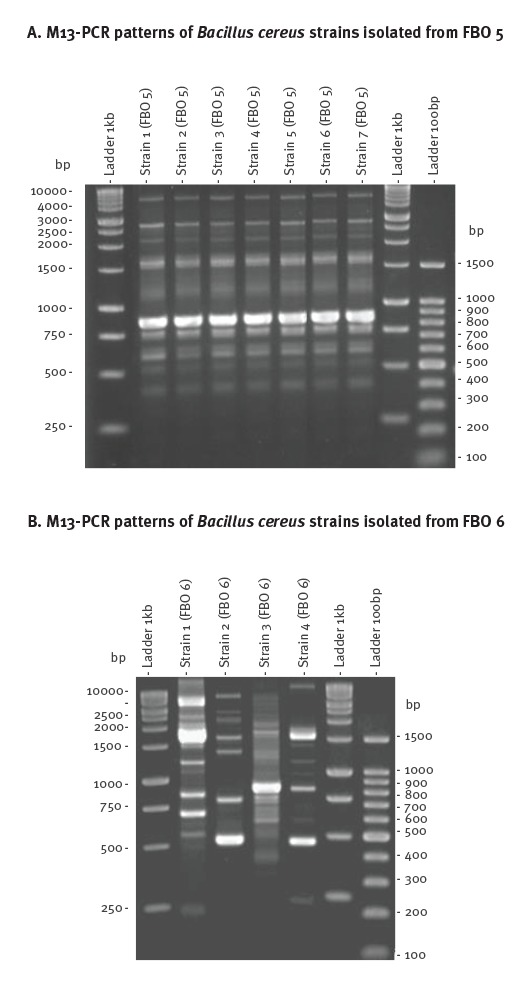
Coliphage M13 sequence-based PCR typing of selected *Bacillus cereus* strains isolated from various samples in two food-borne outbreaks, France, 2007–2014 (n = 11)

**Figure 4:**
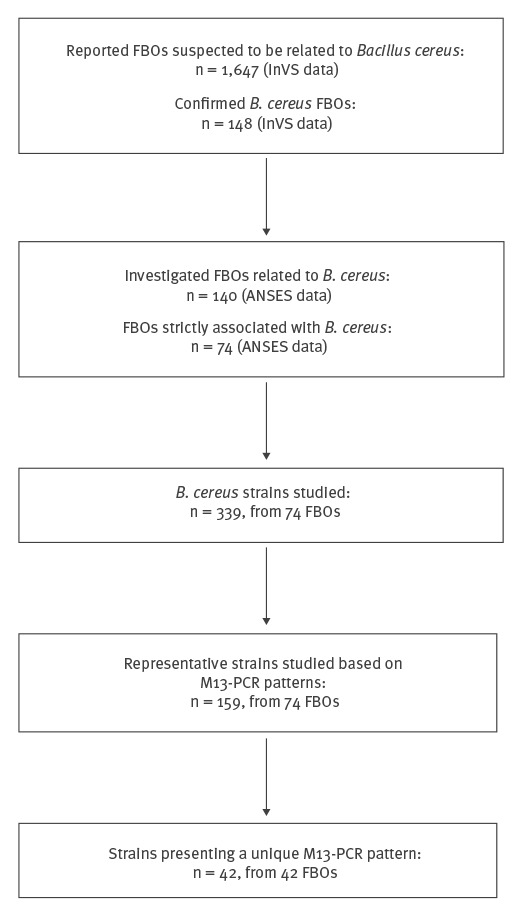
Selection of food-borne outbreaks and panel of *Bacillus cereus* strains studied, France, 2007–2014 (n = 159)

ANSES: French Agency for Food, Environmental and Occupational Health and Safety; FBO: food-borne outbreak; InVS: French Institute for Public Health surveillance, Santé publique France

M13-PCR typing and genetic characterisation were conducted on all 339 *B. cereus* isolates from the 74 FBOs in order to discriminate different patterns and genetic profiles. Up to five isolates from each FBO were subjected to M13-PCR typing. For 42 FBOs, a unique M13 pattern was identified among all isolates recovered from samples within the same FBO (such as FBO number 5, Figure 3A). In the remaining 32 FBOs, several M13 patterns were observed in samples within the same FBO (such as FBO number 6 with four different M13 patterns, Figure 3B). Thus, a total of 159 representative strains gathering 42 strains from the 42 FBOs of unique M 13 pattern and 117 strains representative of the M 13 pattern diversity from the remaining 32 FBOs, were selected for further characterisation (Figure 4).

B: Bacillus; FBO: food-borne outbreak.

The presence of major virulence genes was investigated (Table 2). The *ces* gene was detected in 16% (25/159) of the *B. cereus* strains, meaning they were emetic strains. All the emetic strains belonged to phylogenic group III. The *cytK-1* gene was detected in 5% (8/159) of strains, strictly associated with group VII and classified as *B. cytotoxicus* strains.

The most frequently distributed genes were those encoding enterotoxin Nhe, namely *nheC, nheB* and *nheA* genes detected in respectively 100% (159/159), 99% (157/159) and 96% (153/159) of the tested strains. The *hblA, hblD* and *hblC* genes encoding enterotoxin Hbl were detected in 44% (70/159), 44% (70/159) and 40% (64/159) of the strains, respectively. The c*ytK-2* gene was detected in 42% (67/159) of strains and 23% (37/159) of strains carried *hlyII*.

These genetic features allowed to cluster the strains into 12 pathogenicity or ‘genetic signatures’ (GSs), GS1 to GS12 (Table 2). Some 84% (133/159) of the strains belonged to GS1 to GS6. The most frequent GS encountered in the collection was GS1, which accounted for 21% (34/159) of strains. In GS1, only Nhe-encoding genes were detected. The *ces*-positive strains were all placed in GS3 (except a single one in GS11) and possessed *nhe* genes in addition to the *ces* gene. GS11 also displayed the *cytK-2* gene. GS7 contained all the *B. cytotoxicus* strains carrying the *cytK-1* gene. GS8 was characterised by strains carrying *nheB* and *nheC* genes, and *hblA* and *hblD* genes. All the strains in this group belonged to phylogenetic group VI (Table 2). Several GSs defined in this study were associated with a single *panC* phylogenetic group, i.e. GS2 (IV), GS203 (III) GS7 (VII), GS8 (VI), GS11 (III) and GS12 (II).

## Discussion

Food-borne infections are a common yet distressing and sometimes life-threatening problem for millions of people throughout the world [21]. *B. cereus* is reported to be the fourth major cause of notified FBOs in the European Union and the second in France [4,5]. However, *B. cereus*-associated outbreaks are likely to be underestimated, as they usually remain undiagnosed and therefore under-reported. If *B. cereus* is suspected, several identification tests can be performed: morphology tests on selective media, resistance to polymyxin B, lecithinase synthesis, haemolytic capacity, mannitol fermentation and starch hydrolysis [22]. These tests do not, however, reveal whether the isolated strains are pathogenic nor their genetic features.

The main strengths of our study are the unique national *B. cereus* strain collection linked to strong-evidence FBOs, the long period covered and an accurate epidemiological and strain characterisation. The study of symptoms does not readily allow the identification of the pathogen causing the FBO because gastroenteritis symptoms are also characteristic of other food-borne pathogens, especially *S. aureus* or *C. perfringens* [22]. However, phenotypic analysis and species discrimination allowed us to collect isolates and epidemiological data from 140 FBOs, of which 74 were strictly associated with *B. cereus* and affected 911 human cases. Considering food safety issues, this provides confirmation that *B. cereus* must be considered an important food-borne pathogen, and underlines the need to improve monitoring.

For 32 of these 74 FBOs, several strain patterns were distinguished from samples of a single FBO and it was not possible to discriminate which strain or which combination of strains was responsible for the outbreak, highlighting the need for accurate data on the diversity of the isolated strains during FBO investigation. In contrast, for 42 of the 74 FBOs, a unique strain pattern was identified for each FBO, providing a valuable strain collection for further analysis of the correlation between *B. cereus* genotypic features and associated diseases. Thus, the design of this study strengthens the interpretation of results and avoids bias regarding the bacterial agent causing the FBO.

Our study described 74 FBOs in which only *B. cereus* was recovered. Nevertheless, a limitation of our study is the exhaustivity of the studied FBOs during the period, as the French institute for public health surveillance (InVS, since 2016 Santé publique France) notified 148 FBOs between 2007 and 2014, in which *B. cereus* was the confirmed causative agent (Figure 4). The number FBOs notified to InVS was slightly higher than that of FBOs for which strains were received in ANSES and could be explained by the absence of microbiological investigation of such FBOs or the absence of isolation or sending *B. cereus* strains for further analysis.

Starchy food and vegetables were the most common food vehicles identified in our study. A previous study in commercial cooked chilled foods containing vegetables had shown high *B. cereus* contamination levels in raw vegetables [23]. Thus, particular attention should be taken during sampling and epidemiological investigation into potential *B. cereus* contamination of vegetables and starchy food. In our study, 60% (44/74) of FBOs occurred in an institutional catering context. In the family context, 40% (26/64) of the cases were caused by emetic strains. Incorrect cooling of food during preparation or the conservation of cooked dishes at room temperature is thought to be the cause of cereulide production [24]. Moreover, the severity of symptoms associated with emetic strains might explain an increased reporting of these strains in the family context, compared with diarrhoeic strains which may remain undiagnosed and therefore under-reported.

Epidemiological and clinical data show that the type of symptom could not be specifically associated with the presence of emetic or diarrhoeic strains. Indeed, 57% (n=42) of the 74 FBOs shared both diarrhoeic and emetic syndromes although they were caused by only one type of strain. This may be partially explained by the fact that the emetic GS3 strains strongly produce Nhe enterotoxin (data not shown). We suspect that emetic strains may be ingested concomitantly with cereulide preformed in food, increasing pathogenicity and causing a mix of symptoms.

A significant difference was observed for the incubation period according to the type of strain. This is in accordance with previous findings showing that rapid onset of an emetic syndrome indicates intoxication by cereulide [25]. In contrast, ingestion of diarrhoeic bacteria can induce pathology via the production of enterotoxins in the small intestine, leading to a longer incubation period [26]. In some FBOs, the strains had short incubation periods (0–3 hours) without involvement of emetic strains. We hypothesise that those strains might be responsible for rapid vomiting despite absence of the *ces* gene as previously described [27], or alternatively that the emetic toxin was concomitantly ingested with the contaminated food in addition to a *ces*-negative strain, or that unknown factors were responsible for vomiting symptoms.

Diarrhoeal diseases are often associated with *B. cereus* counts of 10^5^ to 10^8^ cells or spores [28]. In our study, concentrations below 10^3^ CFU/g were found in 12 of 57 foods related to diarrhoeal FBOs. This challenges the concept of a minimum infectious dose for *B. cereus* in diarrhoeal FBOs. A mathematical model based on systematic data collection of *B. cereus* concentrations in food implicated in outbreaks could be developed for dose–response assessment, in order to quantify infectivity associated with single cells [29]. Levels of at least 10^5^ CFU/g have generally been reported in the incriminated food linked to an emetic syndrome [30]. In our study, levels of as few as 400 CFU/g were implicated. This could be explained by cereulide’s strong resistance to various treatments, underlining the importance of quantifying cereulide in foods. We cannot exclude the possibility that the CFUs recovered from leftover food accurately corresponded to the initial ingested CFUs. Indeed, food processing and storage before tests may have injured vegetative bacteria. However, we suspect that the spores, which are resistant to storage, are likely to be responsible for food-borne infections.

The genetic diversity of *B. cereus* strains involved in FBOs was revealed in our study by characterisation of strains based on the detection of the genes encoding cereulide, diarrhoeic toxins (Nhe, Hbl, CytK-1 and CytK-2) and Haemolysin (HlyII) and by phylogenetic classification. A total of 12 pathogenicity signatures based on genetic features of the strains were identified. Emetic strains were clustered in GS3, and possessed both the *ces* gene and the *nhe* genes. This corroborates with the M13 patterns, showing a high clonality of the GS3 group. Surprisingly, all the GS3 strains were unable to hydrolyse starch, although they were mostly found in starchy foods, as published elsewhere [31]. An atypical *ces*-positive strain was classified in GS11, characterised by the presence of the *cytk-2* gene and the absence of Nhe production, despite detection of *nhe* genes (data not shown). This strain was detected once in the analysis of FBO 41, together with a strain belonging to GS4. Such atypical emetic strains have been described [25].

The diarrhoeic strains were more polymorphic than the emetic strains, displaying nine different genetic signatures, although six accounted for 84% (105/125) of the strains. Genes encoding Nhe were present in all GSs, but had variable Nhe production (data not shown), suggesting that other factors may be involved in pathogenicity. GS1 (*nhe* genes only) and GS2 (*nhe*, *hbl* and *cytK2*) were the most prevalent GSs and may have a large impact on human health: they were present in 28% (20/74) and 31% (23/74) of FBOs, respectively. This is consistent with previous findings showing 28% and 24% of *B. cereus* strains belonging to GS1 and GS2, respectively [13]. Unlike GS1 strains, which were divided into three different phylogenetic groups, all GS2 strains belonged to phylogenetic group IV. These strains produce high concentrations of Hbl, are strongly cytotoxic to Caco2 cells and are more prevalent among strains responsible for food poisoning [12]. These characteristics might partially explain the pathogenic potential of strains of GS2, although a synergistic effect of Hbl and Nhe on pathogenicity was not observed [32].

GS7 contained all the *B. cytotoxicus* strains carrying the *cytK-1* gene, which were related to phylogenetic group VII. Strains carrying *cytk-1* were mainly found in vegetable purees, corroborating results of a study showing that 35% of *B. cereus* strains found in commercial potato products taken on retail level or from catering establishments, possess *cytk-1* [33].

Several studies suggest that the pathogenic potential of group VI strains is very low [12]. In our study, these GS8 strains were involved in two FBOs in association with other strains belonging to GS2 and GS7, (FBO 7 and 13, respectively). Thus, it was not proven that GS8 strains were responsible for the symptoms. However, FBO 40, with 18 human cases, was caused by a unique GS8 strain, suggesting a virulence potential of this group [12].

Taken together, assignation of the strains according to genetic signature showed a high genetic diversity of *B. cereus* strains involved in FBOs and their pathogenic potential. Our results underline that *B. cereus* is a food-borne pathogen with a substantial impact on human health that should be investigated when a FBO is suspected. We propose an approach based on reported symptoms and incubation period. Particular attention should be given to vegetables and starchy food during the sampling as part of the investigation. We recommend collecting at least five colonies from each food sample potentially contaminated with *B. cereus*, with different morphologies, as several *B. cereus* with different genetic characteristics may be present in the same food product.
